# Counterfeit and Substandard Test of the Antimalarial Tablet Riamet^®^ by Means of Raman Hyperspectral Multicomponent Analysis

**DOI:** 10.3390/molecules24183229

**Published:** 2019-09-05

**Authors:** Timea Frosch, Elisabeth Wyrwich, Di Yan, Christian Domes, Robert Domes, Juergen Popp, Torsten Frosch

**Affiliations:** 1 Leibniz Institute of Photonic Technology, 07745 Jena, Germany; 2 Friedrich Schiller University, Institute of Physical Chemistry, 07745 Jena, Germany; 3 Friedrich Schiller University, Abbe Centre of Photonics, 07745 Jena, Germany

**Keywords:** Raman spectroscopy, hyperspectral imaging, analytical spectroscopy, counterfeit and substandard pharmaceuticals, DFT calculations, chemometrics, PLSR, API, lumefantrine, artemether, antimalarial tablets

## Abstract

The fight against counterfeit pharmaceuticals is a global issue of utmost importance, as failed medication results in millions of deaths every year. Particularly affected are antimalarial tablets. A very important issue is the identification of substandard tablets that do not contain the nominal amounts of the active pharmaceutical ingredient (API), and the differentiation between genuine products and products without any active ingredient or with a false active ingredient. This work presents a novel approach based on fiber-array based Raman hyperspectral imaging to qualify and quantify the antimalarial APIs lumefantrine and artemether directly and non-invasively in a tablet in a time-efficient way. The investigations were carried out with the antimalarial tablet Riamet^®^ and self-made model tablets, which were used as examples of counterfeits and substandard. Partial least-squares regression modeling and density functional theory calculations were carried out for quantification of lumefantrine and artemether and for spectral band assignment. The most prominent differentiating vibrational signatures of the APIs were presented.

## 1. Introduction

Confidential reports to the World Health Organization (WHO) in the last few years from 20 countries relating to counterfeit drugs revealed that the three highest incidences of fake products were those without active pharmaceutical ingredients (about 30%), followed by incorrect quantities of active ingredients and products with wrong ingredients (about 20% each) [1]. It is estimated that every 10th pharmaceutical product in low- and middle-income countries is substandard or falsified (SF). Antimalarials are the most frequently falsified medicines, representing about 20% of the overall SF products reported in 2017 [2]. Out of the 12 major antimalarial drugs used in the world today, 8 are regularly counterfeited, and more than a third of antimalarial drugs available in sub-Saharan Africa and southeast Asia are reported to be counterfeit or substandard [3].

A report from 2014 [4] showed that among the over 9000 antimalarials sampled, nearly every third failed chemical or packaging quality tests, from which about 40% were classified as counterfeit or substandard and up to 20 wrong active ingredients were found in falsified antimalarials [4].

In 2012 and 2013, one of the most commonly used first-line antimalarials, Riamet^®^, with active pharmaceutical ingredients (APIs) lumefantrine and artemether (also commercialized as Coartem^®^), has been involved in one of the greatest counterfeit scandals of our time. The producing company, Novartis, also officially informed customers of the potential counterfeit “dummy tablets”—without active ingredients—saying “counterfeiting medicines is a serious crime against patients who rely on safe and quality-assured medicines to prevent and cure disease, alleviate pain and save lives” and “reports of adverse reactions […] could materially affect patient confidence in the authentic product, and harm the business of companies such as ours” [5].

Since developing countries are especially concerned of falsified antimalarials, there is an urgent need for low-cost, low-maintenance, easy-to-use, and rapid analytical methods to combat the counterfeit and substandard problem [2]. The Food and Drug Administration (FDA) developed a handheld device named CD-3 [6], which compares scanned images with a stored image of the original product, picking up minute differences in the packaging, pill color, or shape. Although this method is quick and helps to recognize fake packing, it is not chemically selective and does not detect false APIs or false concentrations. Standard techniques, such as high-performance liquid chromatography (HPLC) and mass spectrometry, are highly accurate and reliable, but these methods are strictly lab-based, expensive, time-consuming, and require trained personal. For a quick check, the pH and crystal morphology of the products can be analyzed [3], or a colorimetric test using sulfuric and acetic acid can be applied [7]. This method is based on a color-coded reaction for qualification coupled with color intensity analysis to determine the concentrations of the APIs [7] but chemical selectivity is not ensured.

Raman spectroscopic methods are based on intrinsic molecular vibrations [8,9,10,11,12,13,14] and provide an extremely high chemical selectivity [15,16,17,18,19,20,21,22]. The technique is direct and non-invasive [23,24,25], can be miniaturized, and is also available for on-site applications [26,27,28]. Hence, Raman spectroscopy has already paved its way in counterfeit detection [29,30,31,32,33]. Handheld Raman devices are commercially available from Rigaku Raman Technologies [29], Ahura Scientific, Inc. [30], and B&W Tek, Inc. [32], and all use 785-nm lasers for excitation. These systems are applicable for solid dosage forms. Still, they are not fully reliable for substandard medicine detection and are used as semi-quantitative methods [32]. Another approach for solid pharmaceutical analysis is spatial offset Raman spectroscopy (SORS), where an excitation wavelength also in the near-infrared (NIR) range is applied (824 nm), focusing on the suppression of signals from colored tablets and capsules’ coating [31]. Recently, a line-scanning Raman imaging technique with an excitation wavelength of 785 nm was also reported for API quantification [33].

In this work, we present a proof-of-principle study using fiber-array based Raman spectroscopy [34] with an excitation wavelength in the visible range (532 nm) for multicomponent concentration analysis and counterfeit testing of the antimalarial tablet Riamet^®^.

Our method allows us to reliably qualify and quantify the active ingredients lumefantrine and artemether in tablets without dissolving them, as it is done for the standard HPLC analysis. By using an 8 × 8 fiber array, 64 spectra can be collected simultaneously, thus analyzing a larger area of the tablets is possible with only one measurement in a time-efficient way. This advantage is of great importance, as pharmaceutical samples are often heterogeneous. By illuminating the sample surface with a bigger field-of-view (FOV) instead of a mere spot, variations of the spatial concentration distribution can be visualized. The fiber array imaging setup presented here operates with an excitation wavelength of λ = 532 nm, thus the Raman scattering intensity is enhanced in comparison to excitation wavelengths in the NIR according to Equation (1), where *N* is the number of scatterers, *I*_0_ is the laser intensity, *ν*_0_ is the frequency of the excitation laser, and *α* is the polarizability of the molecule. This offers the chance to quantify substandard drugs with lower amounts of API.

(1) $$I_{\mathrm{STOKES}}\propto N\cdot I_{0}\cdot {\left(\nu_{0}-\nu_{r}\right)}^{4}\cdot {\left|\alpha \right|}^{2}.$$

## 2. Results and Discussion

**Figure 1:**
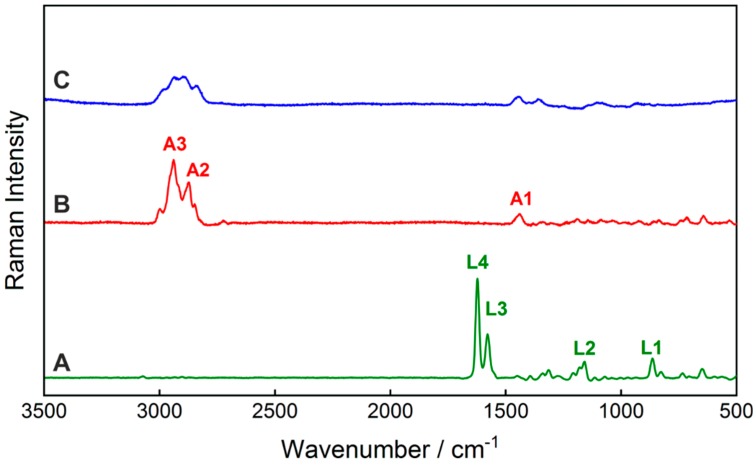
Raman spectra of the active pharmaceutical ingredients lumefantrine (**A**) and artemether (**B**), as well as the excipient hypromellose (**C**), with an excitation wavelength of λ = 532 nm. The spectra of artemether and hypromellose were scaled with a factor of five for better visibility. The band assignment of the prominent Raman bands A1–A3 and L1–L4 and their spectral positions are listed in Table 1.

This work reports the simultaneous qualification and quantification of two APIs in a pharmaceutical tablet by means of fiber array-based hyperspectral Raman imaging for the first time. First, the Raman spectra of the pure tablet ingredients, lumefantrine, artemether, and hypromellose, were acquired (Figure 1). The vibrational band assignments of the active ingredients were performed based on density functional theory (DFT) calculations and are summarized in Table 1. A comparison of the calculated Raman spectra with the experimentally acquired FT-Raman spectra confirmed a very good agreement (Figure S1). The characteristic Raman bands of lumefantrine were assigned to the vibrational modes from the benzene ring stretching (L3), C=C stretching (L4), and CH deformational vibrations (L1, L2). The dominant Raman bands of artemether were mostly assigned to different CH vibrations (A1—CH_3_ wagging, A2—asymmetric stretching of CH_2_ combined with slight CH-stretching, A3—asymmetric CH_2_ stretching). The latter ones overlap with the Raman modes of the excipient hypromellose. The quantification of artemether in the presence of hypromellose is therefore challenging. To qualify and quantify the APIs lumefantrine and artemether based on the Raman spectra of the tablets in a reliable way, it is necessary to apply multivariate data analysis approaches. A very robust quantitative chemometric method is partial least squares regression (PLSR).

### 2.1. Development of Partial Least-Squares Regression Model for Ingredient Quantification

Spectral preprocessing is an essential part of modeling to increase the accuracy of the predictions by reducing influences that account to noise-related signal contributions. First, a fiber intensity correction was applied on the hyperspectral image data of the pure substances lumefantrine, artemether, and the model tablets Lu100Ar100, Lu50Ar100, Lu100Ar0, and Lu0Ar100. Afterwards, unit vector normalization was used to correct for Raman intensity variations due to technical effects like different optical path lengths or sample density variations, etc. [35] followed by Savitzky–Golay smoothing. Multiplicative scatter correction (MSC) was section-wise applied for an expanded baseline correction to reduce Raman intensity variations due to different particle sizes [36].

PLSR combines a factorial analysis and a regression method. First, a PLSR calibration model was built, considering simultaneously the responses from the analytes, such that the concentrations exactly summed up to 100% (PLS2 approach). Afterwards, the PLSR calibration model was applied to the hyperspectral images of the model tablets. For validation of the model, external validation is preferred [37]. In case of hyperspectral images, it was possible to use one half of the image for calibration and the other half for validation [38]. However, this approach was not beneficial in the case of the tablets, as they are heterogeneous, and thus the spatial variations of concentrations did not match the input reference values for the model development. Influences caused by outliers and heterogeneities can be reduced by summarizing a single hyperspectral image as a median spectrum. To build up a representative data set for calibration and validation, the Kennard–Stone algorithm was applied in combination with a prior cross validation to remove outliers that would otherwise be taken as extreme samples [39,40]. A good correlation between the predicted and reference data for both the calibration (R^2^ = 0.9829 for lumefantrine and R^2^ = 0.9989 for artemether) and for the validation PLSR-model (R^2^ = 0.9827 for lumefantrine and R^2^ = 0.9982 for artemether) was achieved. The predictive error for the validation (RMSE) were 5.00 wt% for lumefantrine and 1.59 wt% for artemether.

### 2.2. Active Ingredient Concentration Prediction and Interpretation of the Spectral Information of the Model

The prediction model was applied to 30 hyperspectral images of each model tablet and for the Riamet^®^ tablet, respectively. The predicted concentrations and the corresponding error ranges are listed in Table 2. The occurrence of outliers was reduced by using median-averaged images.

The predicted mean concentrations for lumefantrine were found around the expected 60 wt%, (4.5–7.7 wt% deviation) (Table 2). For the substandard tablet Lu50Ar100 (containing 50% of the nominal lumefantrine and 100% of the artemether content), the predicted mean concentration was above the expected one, whereas for Riamet^®^ it was 5 wt% below the expected value (Table 2). For artemether, the predicted concentrations fitted very well to the expected ones, deviating only 0.5 to 1.5 wt% in the content of the model tablets and 2.7 wt% in the case of Riamet^®^ (1.4–4.7 wt% deviation) (Table 2). The United States Pharmacopoea requires at least 30 samples for the content uniformity test and allows a maximum range of 25% for deviation from the reference value of a single dosage unit tested [41]. Thus, our observed deviations are well covered in this range. The observed deviations from the expected values are partly caused by the inhomogeneous scattering effects of the surface, combined with limited signal-to-noise ratios, and partly with the uncertainty of the regression model (RMSE of prediction are 5.00 wt% for lumefantrine and 1.59 wt% for artemether). It should also be noted that for the model, the target wt% values in the training group were defined based on the nominal added amounts of the ingredients. This can also lead to some minor errors in the prediction. Lumefantrine is a strong Raman scatterer, and the absolute Raman signal variations of the different concentrations of lumefantrine are much higher than those of artemether. Hence, their simultaneous quantification requires a compromise in the accuracy of the predictions.

**Figure 2:**
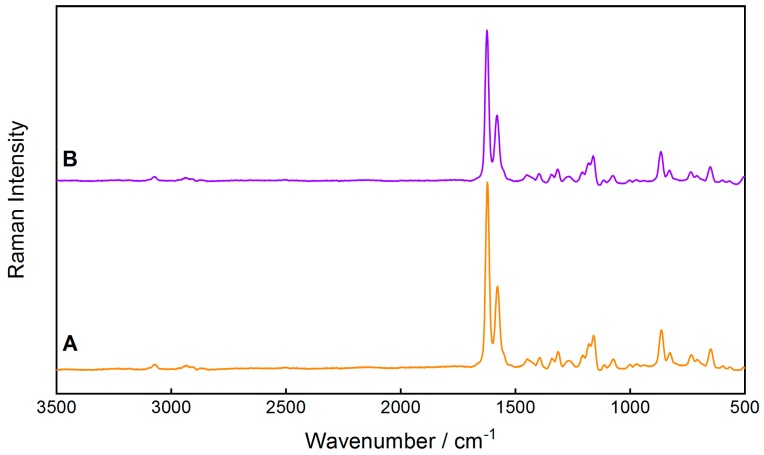
Comparison of the Raman spectra of (**A**) the genuine Riamet^®^ tablet and (**B**) the model tablet Lu100Ar100 with the nominal 100% content of the active ingredients.

For better prediction accuracy for the genuine tablet Riamet^®^, it would be beneficial to include more excipients in the calibration and validation model. Only hypromellose was used as an excipient, but microcrystalline cellulose, croscarmellose sodium, magnesium stearate, polysorbat 80, and highly dispersed SiO_2_ were not considered in the calibration model. As the producing company does not share such detailed information on the exact composition of the tablets, this aspect remains challenging. However, the comparison between the Raman spectrum of the model tablet Lu100Ar100, containing the full content of the APIs lumefantrine and artemether, with the spectrum of the genuine Riamet^®^ tablet show a high similarity (Figure 2) and justifies this approximation.

**Figure 3:**
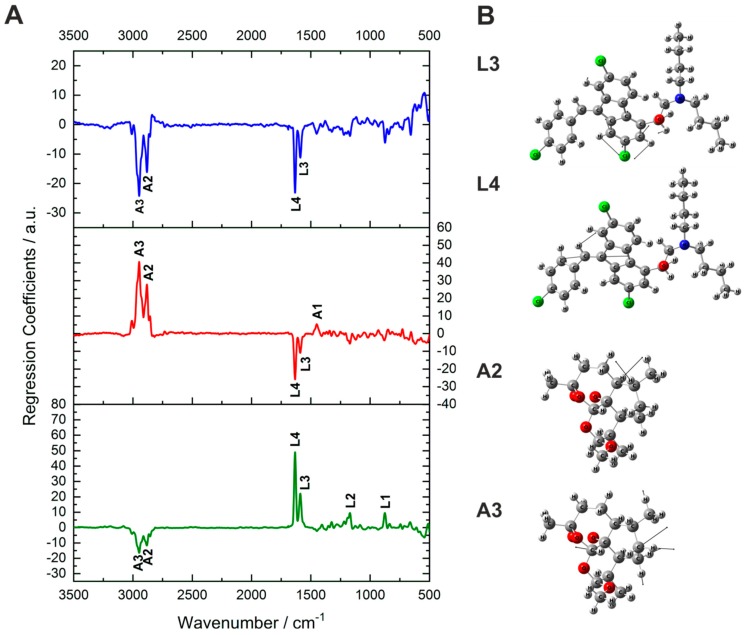
(**A**): Regression coefficients for the prediction of lumefantrine (green, lower part), artemether (red, middle part), and hypromellose (blue, upper part). The coefficients from the first two factors for each analyte correlate perfectly to the characteristic Raman bands of lumefantrine and artemether. Strong contribution for the differentiation is attributed to the peaks L4, L3, A3, and A2. (**B**): Vibrational assignment of the peaks that contribute most to the PLSR model: L3: benzene ring stretching + CH scissoring, L4: C=C stretching vibration + CH scissoring, A2: asymmetric stretching vibration + slight contribution from CH stretching, A4: asymmetric CH_2_ stretching vibration.

The most-representative Raman bands of the active ingredients correlate well to the large regression coefficients (Figure 3A), which account for a high influence of the respective Raman signal in the prediction. The prominent Raman bands of both lumefantrine and artemether correlate with high positive coefficients of their own prediction factors (especially L3 and L4 and A2 and A3). This underlines that the model differentiated correctly between the active ingredients based on the respective spectral information.

Hypromellose and artemether have their strongest Raman bands in the same spectral regions between 2800 and 3000 cm^−1^ and some spectral overlap occurs. Nevertheless, the developed model enabled the quantification of artemether in the presence of hypromellose. This is demonstrated by the high negative coefficients for the prediction of hypromellose at the positions of A2 and A3 (Figure 3A). For better visualization of the molecular information underneath the Raman bands, the vibrational assignments of Raman bands L4, L3, A3, and A2 are depicted (Figure 3B). L3 is a combination of a benzene ring stretching and CH scissoring of lumefantrine. L4 is a C=C stretching vibration combined with a less-intensive CH scissoring of lumefantrine. A2 is an asymmetric CH_2_ stretching vibration with a slight contribution from CH stretching of artemether, whereas A3 is an asymmetric CH_2_ stretching vibration of artemether.

**Figure 4:**
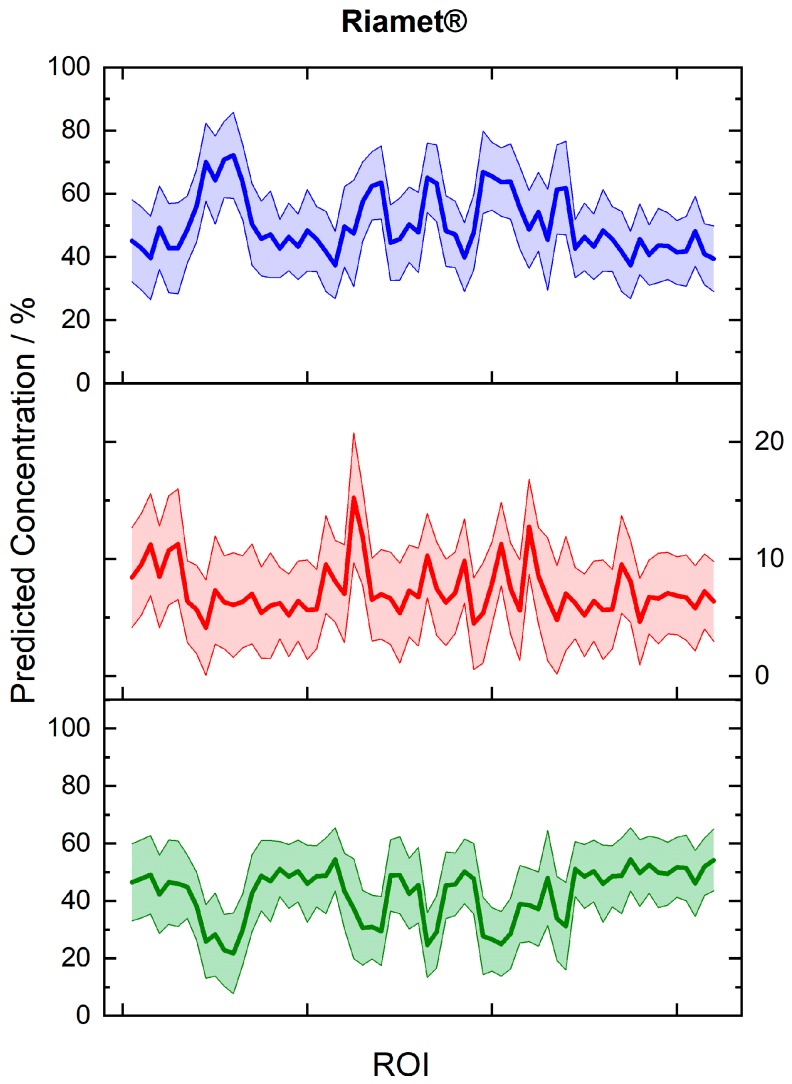
Predicted concentrations for 64 random spots from 30 regions (30 hyperspectral images) of the three constituents in Riamet^®^: lumefantrine (green line, lower graph), artemether (red line, middle part), and hypromellose (blue line, upper graph). The respective prediction error ranges are shown. Local differences in the distribution of the concentrations of active ingredients in the tablet are revealed. Each region of interest (ROI) indicates the imaged area from a single fiber in the fiber array.

**Figure 5:**
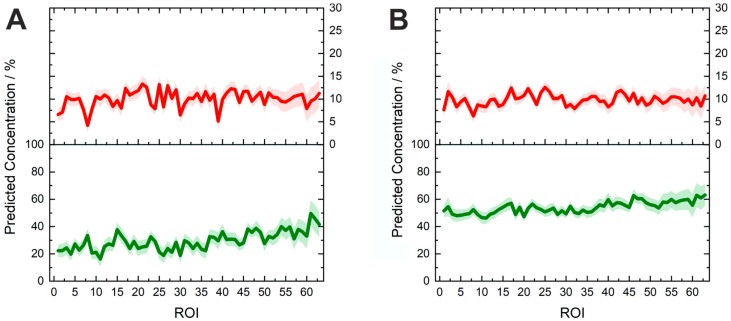
Predicted concentrations of lumefantrine (green, lower line) and artemether (red, upper line) in different spots on the model tablets. Each region of interest (ROI) indicates the imaged area from a single fiber in the fiber array. (**A**) Lu50Ar100: 50% of the nominal lumefantrine and 100% of the nominal artemether content, corresponding to 30 wt% lumefantrine and 10 wt% artemether in the tablet. (**B**) Lu100Ar100: 100% the nominal content of lumefantrine and artemether, corresponding to 60 wt% lumefantrine and 10 wt% artemether in the tablet).

The predicted concentration values and the corresponding uncertainty ranges of Riamet^®^ were presented for 64 random regions from 30 hyperspectral images (Figure 4). Differences of the API concentrations in different parts of the tablets were revealed. For lumefantrine, the local concentrations varied between 21.8 and 54.5 wt% and for artemether between 4.1 and 15.2 wt%, most probably due to an inhomogeneous API distribution. The active ingredients in the model tablets were more homogenously distributed (Figure 5). It is easily obvious that the model tablet with 50% of the nominal lumefantrine and 100% of the artemether content (Figure 5A) has a lower lumefantrine content than the one with a full nominal content (Lu100Ar100) (Figure 5B), as it was expected. This demonstrates the suitability of the presented method to gain information about substandard tablets directly and non-invasively (without dissolution). The concentrations varied on the spot level between 16.1 and 49.6 wt% in the substandard model Lu50Ar100, which corroborates the necessity of acquiring data over numerous areas of pharmaceutical tablets. This can be done in a very time-efficient manner with the presented fiber array-based Raman imaging technique, which allows the simultaneous measurement of 64 sample spots with one measurement. Furthermore, local concentration variations can also be easily visualized (Figure 5), which will be an extremely helpful ability in non-invasive quality control of tablets.

### 2.3. Potential of Fiber Array-Based Technique for Counterfeit and Substandard Tablet Testing

The fiber array-based Raman hyperspectral imaging technique provides the following advantages, which can be exploited for counterfeit and substandard testing of pharmaceutical tablets: The presented method is non-invasive and non-destructive, without using any aggressive or toxic solvents. Thus, this method is environment-friendly and cost-effective.

Combining Raman measurements with chemometric modeling, both qualitative and quantitative information of several analytes are captured in one single measurement procedure, granting high potential for the efficient investigations of pharmaceutical samples to detect low-quality issues. Using a high magnification objective with a high NA additionally allows visualization of the API distribution in a highly resolved way (e.g., lumefantrine in Figure S2A). Another strong advantage is the time-efficient measurement procedure, as 64 Raman spectra can be acquired simultaneously (Figure S2B). The setup presented in this proof-of-principle study is flexible and can adapt to different experimental settings, as the amount of collected spectra in one shot can be further extended using different fiber array configurations and the dimensions of the FOV at the sample can easily be changed.

## 3. Materials and Methods

### 3.1. Chemicals and Tablets

**Figure 6:**
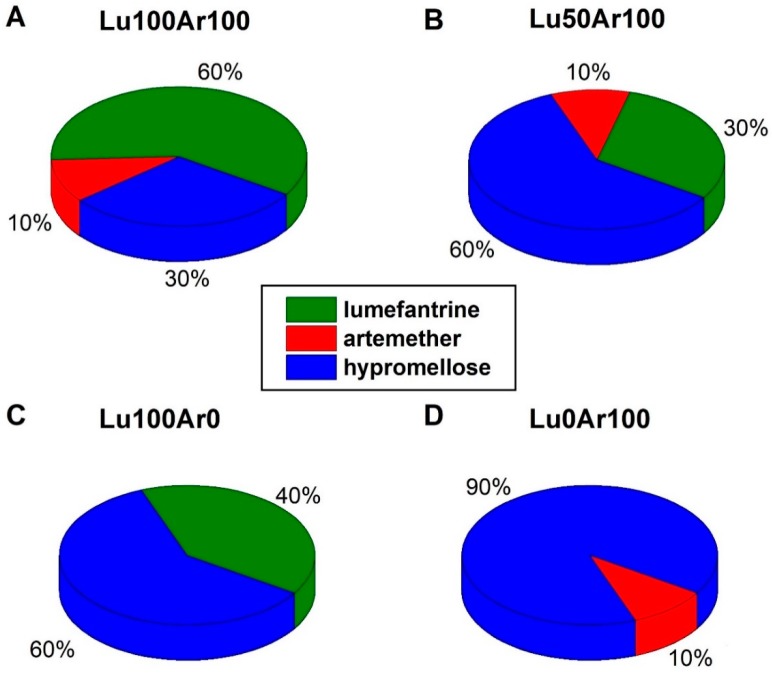
Composition of the anti-malarial model tablets. 100% refers to the nominal content in the original Riamet^®^ tablet, which are 120 mg lumefantrine and 20 mg artemether, corresponding to 60 wt% lumefantrine, 10 wt% artemether, and 30 wt% filling excipient hypromellose in the tablet. The total mass of each tablet is 200 mg. (**A**) Lu100Ar100: Content of nominal 100% lumefantrine and nominal 100% artemether (60 wt% lumefantrine, 10 wt% artemether and 30 wt% filling excipient hypromellose in the tablet). (**B**) Lu50Ar100: Content of nominal 50% lumefantrine and nominal 100% artemether (30 wt% lumefantrine, 10 wt% artemether, and 60 wt% filling excipient hypromellose in the tablet). (**C**) Lu100Ar0: Content of nominal 100% lumefantrine and nominal 0% artemether content (40 wt% lumefantrine and 60 wt% filling excipient hypromellose in the tablet). (**D**) Lu0Ar100: Content of nominal 0% lumefantrine and nominal 100% artemether content (10 wt% artemether and 90 wt% filling excipient hypromellose in the tablet).

Lumefantrine (Lu), artemether (Ar), and hypromellose were purchased from Sigma Aldrich (Taufkirchen, Germany). Model fake tablets were manufactured, containing the APIs lumefantrine and artemether in different concentration ratios by direct compression. The total weight for each model tablet was 200 mg and the pharmaceutical excipient hypromellose was used to fill up the formulation. The composition of the analyzed tablets is visualized in Figure 6. Riamet^®^ tablets (Novartis) were purchased from a local pharmacy (Jena, Germany) and investigated. The coating of this tablet was removed for better conformity with the model tablets.

### 3.2. FT-Raman Spectroscopy

The FT-Raman spectra of the active ingredients lumefantrine and artmether were recorded using a Bruker FT-Raman spectrometer (Ram II) (Bruker Optik GmbH, Germany) with an Nd:YAG laser operating at 1064 nm. The spectral resolution was set to 4 cm^−1^.

### 3.3. Density Functional Theory Calculation

To better assign and interpret the Raman bands of the active ingredients, the vibrational modes and Raman scattering activities were calculated with the help of density functional theory (DFT) using Gaussian 16 [42]. The hybrid exchange correlation functional with Becke’s three-parameter exchange functional (B3) [43] slightly modified by Stephens et al. [44] coupled with the correlation part of the functional from Lee, Yang, and Parr (B3LYP) [45] and Dunning’s triple (cc-pVTZ) correlation consistent basis sets of contracted Gaussian functions with polarized and diffuse functions [46] at standard conditions were applied. Separate scaling factors for the lower (<2000 cm^−1^) and for the higher (>2000 cm^−1^) wavenumber regions and an intensity correction were estimated [13,47].

### 3.4. Fiber-Array Based Hyperspectral Imaging

**Figure 7:**
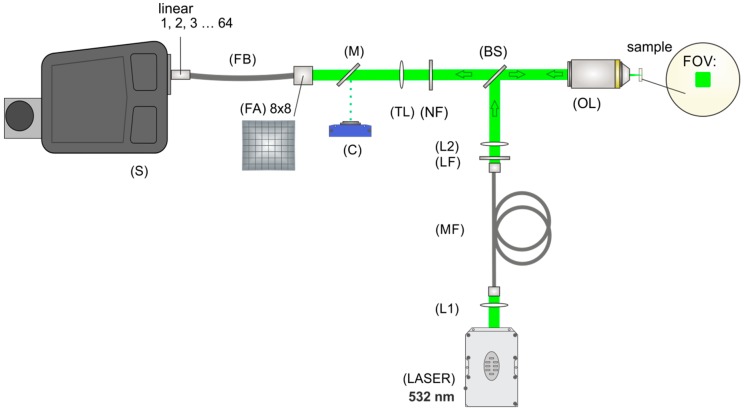
The experimental setup for fiber array-based Raman hyperspectral imaging is divided into an illumination and an imaging part, separated by a beam splitter (BS). The illumination part consists of a laser for excitation (LASER), two lenses (L1 and L2), a step index multimode fiber (MF), a cleanup filter (LF), and an objective lens (OL). Light is scattered back from the sample, collected by the same objective lens (OL), and imaged with the help of a tube lens (TL) onto the entrance face of a fiber array (FA). A suitable sample region can be chosen by directing the light onto a camera (C) with the help of a flip mirror (M). A notch filter (NF) removes the laser excitation wavelength and elastically scattered light. The scattered light is collected by the 8 × 8 array and is transformed with the help of a specially designed fiber bundle (FB) into a linear fiber array at the distal end and positioned in the slit plane of the spectrometer (S).

The spectroscopic measurements of the samples (the powder form APIs lumefantrine and artemether, the excipient hypromellose, the model tablets, and Riamet^®^) were carried out with a hyperspectral imaging setup. The sample area was illuminated with an FOV of 10 × 10 µm^2^ (Figure 7). The laser power in the sample plane was 600 mW and an exposure time of 10 s was used with three accumulations. A specially designed fiber-array bundle was applied for signal collection (Figure 7). The sample surface was imaged onto the entrance face of the fiber array and the shape of the bundle was transformed from an 8 × 8 square to a linear array of 64 fibers. The line of fibers was then placed in the plane of the spectrometer slit (IsoPlane, Princeton Instruments) and enabled the simultaneous acquisition of 64 spectra (Figure 7). After the acquisition of the spectra, pre-processing tools, such as baseline correction (rolling-ball algorithm) and spike correction, were applied using LabVIEW. To provide a representative spectrum of the tablets Riamet^®^ and the model tablet Lu100Ar100 (Figure 2), 10 hyperspectral images per tablet were acquired and for each image the median spectrum was calculated. From the 10 median spectra, an average spectrum was calculated, and a second baseline correction was carried out with the SNIP algorithm (2nd order). Each spectrum was assigned to a specific spot in the sample area and hyperspectral images were built based on the desired chemical information.

### 3.5. Partial Least-Squares Regression Model for the Ingredients’ Quantification

For the spectral analysis and modeling, the chemometrics software ‘The Unscrambler^®^ X 10.3’ (Camo Software AS., Oslo, Norway) was used.

## 4. Conclusions

In this work a proof-of-principle study using a novel method to qualify and quantify substances in pharmaceutical tablets that are potentially counterfeit or substandard was presented. Based on a fiber array-based Raman hyperspectral imaging technique combined with PLSR modeling, the concentrations of the APIs lumefantrine and artemether were simultaneously determined in model tablets and in the tablet Riamet^®^. The analysis was carried out in a non-destructive way, without dissolution, which is an advantage in comparison to conventional methods. In addition, the concentration distribution of active ingredients could also be assessed. Being able to identify and quantify counterfeits (Lu100Ar0, Lu0Ar100) and even substandard (Lu50Ar100) antimalarial tablets fast and directly on the tablet gives us a new tool for the fight against falsification of pharmaceuticals. The analyzed tablet Riamet^®^ is of high importance, since antimalarial tablets are the most frequent targets of counterfeiting in the world, as highlighted by the WHO and the FDA.

In future work, we intend to test “real fake” samples, thus complementing our training model. It would be highly beneficial to apply the presented easily applicable and flexible technique as a first test to detect peculiarities or abnormalities before analyzing the tablets with destructive and more expensive analytical techniques.

In summary, fiber array-based Raman hyperspectral imaging in combination with PLSR analysis enables a fast and chemically selective, noninvasive, and spatially resolved determination of multicomponent API concentrations in pharmaceutical tablets, showing high potential as a future “anti-fake and substandard tool”.

## Figures and Tables

**Table 1:** Band assignment of the prominent Raman peaks of lumefantrine and artemether.

Lumefantrine					Artemether				
Peak Position/cm^−1^					Peak Position/cm^−1^				
Identification	Measured 532 nm	Measured FT-Raman	Calculated *	Band Assignment	Identification	Measured 532 nm	Measured FT-Raman	Calculated *	Band Assignment
**L1**	865	876	875	δCH + ωCH	**A1**	1442	1454	1455	δ_s_CH_3_
**L2**	1180	1172	1170	δCH	**A2**	2872	2873	2874	ν_as_CH_2_ (+ νCH)
**L3**	1580	1589	1587	νb + δCH	**A3**	2940	2937	2937	ν_as_CH_2_
**L4**	1623	1635	1640	νC=C + δCH					

ν_s_: symmetric stretching vibration; ν_as_: asymmetric stretching vibration; δ: scissoring; δ_as_ asymmetric bending vibration (twisting); δ_s_: symmetric bending vibration (wagging); ω: bending vibration; ν_b_ (benzene ring stretching); * For the band position assignments based on the DFT calculations different scaling factors were applied: 0.98 for the spectral regions below 2000 cm^−1^ and 0.95 for the region above 2000 cm^−1^.

**Table 2:** Predictions of the lumefantrine and artemether concentrations in the model tablets and the genuine tablet as follows: Lu100Ar100 (100% nominal lumefantrine and 100% artemether content, corresponding to 60% lumefantrine and 10% artemether in the tablet), Lu50Ar100 (50% nominal lumefantrine and 100% artemether content, corresponding to 30% lumefantrine and 10% artemether in the tablet), Lu100Ar0 (100% nominal lumefantrine and 0% artemether content), and Lu0Ar100 (0% nominal lumefantrine and 100% artemether content) based on the partial least squares regression (PLSR) model.

Tablet	Lumefantrine Concentration/wt%			Artemether Concentration/wt%		
	Expected	Predicted	*y*_dev_	Expected	Predicted	*y*_dev_
**Lu100Ar100**	60.0	57.8	4.5	10.0	9.5	1.4
**Lu50Ar100**	30.0	44.1	6.1	10.0	9.1	1.9
**Lu100Ar0**	60.0	59.8	7.7	0.0	1.0	2.4
**Lu0Ar100**	0.0	1.2	6.3	10.0	11.4	2.0
**Riamet^®^**	50.0	44.1	14.6	8.3	5.6	4.7

$y_{\mathrm{dev}}$ describes the deviation of the concentration prediction.

## Supplementary Materials

The following are available online at https://www.mdpi.com/1420-3049/24/18/3229/s1, Figure S1: Comparison of the calculated Raman spectra (DFT) with the experimentally acquired FT-Raman spectra of the active ingredients lumefantrine and artemether. Figure S2: Exemplary visualization of the spatial distribution of the lumefantrine concentration along one hyperspectral image in the model tablet Lu100Ar100.
