# Supplementary material for: Counterfeit and Substandard Test of the Antimalarial Tablet Riamet® by Means of Raman Hyperspectral Multicomponent Analysis
Source: Molecules. 2019 Sep 5;24(18):3229. doi: 10.3390/molecules24183229 (PMC6767462; doi:10.3390/molecules24183229)
Supplement: Supplementary file 1 [file molecules-24-03229-s001.pdf]

Supporting Information

Counterfeit and substandard test of the antimalarial tablet Riamet® by means of Raman hyperspectral multicomponent analysis

Timea Frosch[1°], Elisabeth Wyrwich[1°], Di Yan[1], Christian Domes[1], Robert Domes[1], Jürgen Popp[1,2,3] and Torsten Frosch[1,2,3,*]

[1]Leibniz Institute of Photonic Technology, Jena, Germany

[2]Friedrich Schiller University, Institute of Physical Chemistry, Jena, Germany

[3]Friedrich Schiller University, Abbe Centre of Photonics, Jena, Germany

[°]both authors contributed equally

[*]corresponding author: torsten.frosch@uni-jena.de, torsten.frosch@gmx.de,

ORCHID: 0000-0003-3358-8878

Figure S1: Comparison of the calculated Raman spectra (DFT) with the experimentally acquired FT-Raman spectra of the active ingredients lumefantrine and artemether

Figure S2: Exemplary visualization of the spatial distribution of the lumefantrine concentration along one hyperspectral image in the model tablet Lu100Ar100

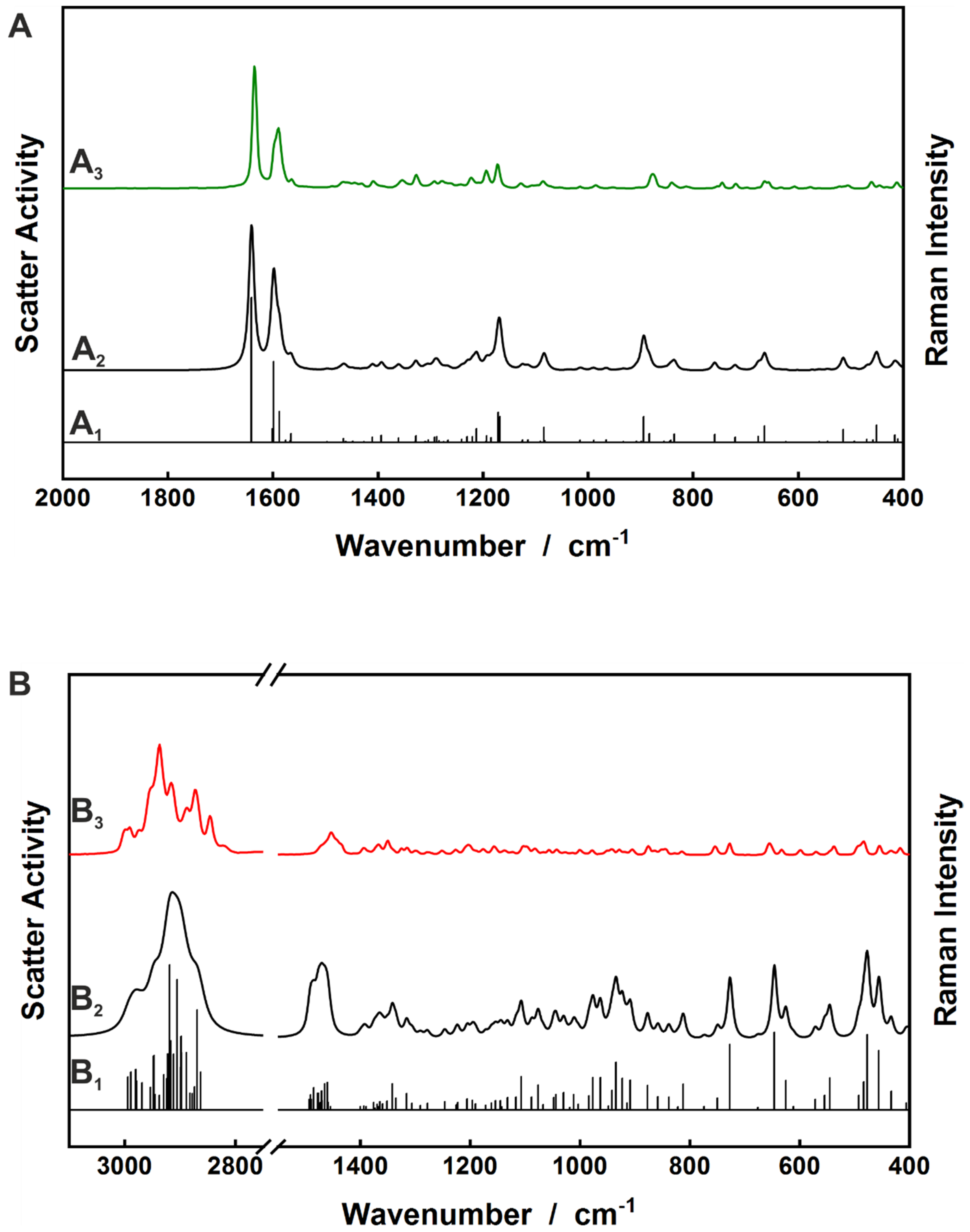

Figure S1: Comparison of the calculated Raman spectra (DFT) of the active ingredients lumefantrine (**A$_2$**) and artemether (**B$_2$**) with their experimentally acquired FT-Raman spectra (**A$_3$** and **B$_3$**). **A$_1$** and **B$_1$** depict the calculated Raman scattering activities, applying the scaling factors 0.98, for the spectral regions below 2000 cm^{-1}, and 0.95 for the region above 2000 cm^{-1}.

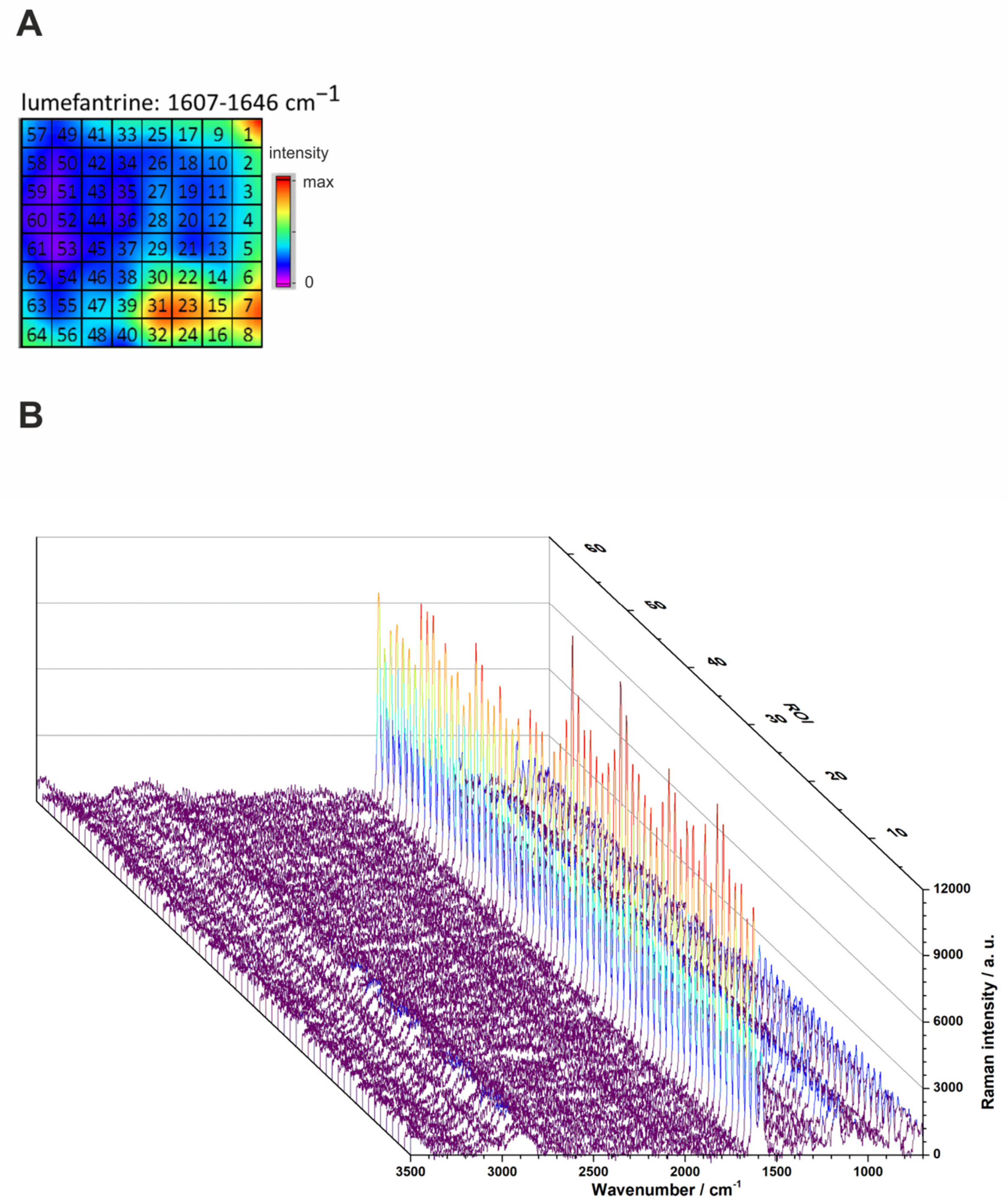

Figure S2: Exemplary visualization of the spatial distribution of the lumefantrine concentration in the model tablet Lu100Ar100 (based on the wavenumber range: 1607-1646 cm^{-1}). (**A)** The numbers 1 to 64 indicate the regions of interest (ROI) for the single fibers in the 8x8 fiber array. (**B)** Raman hyperspectral image consisting of 64 single Raman spectra with varying intensities due to spatial inhomogeneities of the APIs.
